# Supplementary material for: Risk-taking unmasked: Using risky choice and temporal discounting to explain COVID-19 preventative behaviors
Source: PLoS One. 2021 May 13;16(5):e0251073. doi: 10.1371/journal.pone.0251073 (PMC8118306; doi:10.1371/journal.pone.0251073)
Supplement: S1 File — (DOCX) [file pone.0251073.s001.docx]

**S1 File.**

Risk-Taking Unmasked:

Using Risky Choice and Temporal Discounting to Explain COVID-19 Preventative Behaviors

Kaileigh A. Byrne, Stephanie G. Six, Reza Ghaiumy Anaraky, Maggie W. Harris, & Emma L. Winterlind

*Clemson University*

**Regression Results that Include Prosocial Behavior**

To determine whether prosociality influenced the outcome measures, correlational analyses between the two prosocality measures (the Prosocial Behavioral Intentions Scale and Dictator Game) and the outcome measures were performed within the second wave of data collection alone (*n*=197). Table S9 shows these results. Higher scores on the Dictator Game, indicative of greater prosocial behavior, were associated with *increased* participation in interpersonal social interactions (*r* = .160, *p* = .025) and engagement in non-essential activities (*r* = .196, *p* = .006). No other significant correlations were observed (*p*s>.05).

Moreover, supplemental linear regression analyses that included scores on the Dictator Game were performed for each outcome measure. Thus, the supplemental regressions included Proportion of Risky Choice Behavior, Temporal Discounting, Difference in Perceived Risk, and Dictator Game score as predictors as well as the covariates described in the main text. Results showed that prosocial behavior as indexed by the Dictator Game was a significant *positive* predictor of engaging in non-essential activities (β = .178, *p*=.004) but was not significantly predictive of Mask-Wearing Behavior (β = -.064, *p*=.329), Interpersonal Social Interactions (β = 1.26, *p*=.065), or Perceived Risk (β = .055, *p*=.415), nor did the inclusion of this variable affect the significance level of Temporal Discounting or Proportion of Risky Choices in these analyses. Thus, with the inclusion of the Dictator Game prosocial behavior variable in the regression, Temporal Discounting and Proportion of Risky Choices were still significantly predictive of mask-wearing and social distancing. Prosocial behavior was also not predictive of the Optimism Bias (β = -.104, p=.155), but when this measure was included in the analysis, risky choice (β = -.075, *p*=.334) was no longer predictive of the optimism bias.

**Multivariate Regression**

A supplemental multivariate regression that included all independent variables (Temporal Discounting and Proportion of Risky Choices) and dependent variables (Appropriate Mask-Wearing, Interpersonal Social Interactions, Non-Essential Social Activities, Optimism Bias, and Perceived Risk) was performed. Results largely mirrored the findings for the linear regressions reported in the main text. Temporal Discounting and Proportion of Risky Choices predicted Appropriate Mask-Wearing, Interpersonal Social Interactions, and Non-Essential Social Activities (*p*s<.001). The results are reported in Table S10.

**Exploratory One-Way ANOVAs for Political Affiliation**

While the regression results did not reveal a significant effect of political affiliation on mask wearing and social distancing, several recent studies have found evidence that political affiliation is predictive of compliance with mask-wearing and social distancing recommendations. Based on this prior work, exploratory one-way ANOVAs were conducted for political affiliation predicting mask-wearing and social distancing to gain better insight into these potential relationships. One-way ANOVA results for Political Affiliation predicting Appropriate Mask Wearing was significant, *F*(2, 401)=16.64, *p*=.001, and post-hoc tests revealed that Republicans (*M*=64.49, *SD*=40.70) reported less mask-wearing than Democrats (*M*=84.98, *SD*=29.57) and Independents (*M*=85.31, *SD*=28.39). Similar results for Interpersonal Social Interactions (*F*(2, 401)=19.81, *p*=.001) and Engagement in Non-Essential Public Activities (*F*(2, 401)=16.63, *p*=.001) were observed in which Republicans reported significantly more mask-less interpersonal interactions and non-essential public activities. Additional one-way ANOVAs were performed for Proportion of Risky Choices. Surprisingly, the one-way ANOVA for Political Affiliation predicting risky choices was significant, *F*(2, 401)=6.845, *p*=.001, and the post-hoc follow-up tests indicated that Republicans (*M*=0.245, *SD*=0.216) made more risky choices on the task than Democrats (*M*=0.197, *SD*=0.184) or Independents (*M*=.149, *SD*=.165). Similarly, Republicans (*M*=.534, *SD*=.293) had higher delay discounting scores than Democrats (*M*=.629, *SD*=.286) or Independents (*M*=.613, *SD*=.283), *F*(2, 401)=4.301, *p*=.014. These findings tentatively suggest that while political affiliation alone is associated with differences in mask-wearing and social distancing, covariance with Risk-Taking and Delay Discounting may mask these effects.

**Table S1.**

| COVID-19 Social Distancing Behaviors | | |  |  |
| --- | --- | --- | --- | --- |
|  | ***Mean*** | ***SD*** | | |
| Number of people participants had face-to-face interactions with *without*  wearing masks/social distancing in the past 14 day | 4.02 | 6.49 | | |
| Number of people that participants had face-to-face interactions  with *while* wearing masks or social distancing in the past 14 days | 8.11 | 9.03 | | |
| Times where participants spent time in a group of 20+ people | 2.66 | 6.60 | | |
| Times participants spent time in a group of 3 – 5 people | 3.56 | 6.90 | | |
| Times participants left home for reasons other than work | 6.25 | 6.58 | | |
| Times participants ate at a dine-in restaurant in the past 30 days | 2.36 | 5.97 | | |
| Times participants went to the mall/shopping center in past 30 days | 3.08 | 6.37 | | |
| Times participants went to the hair salon, nail salon, or  barbershop within the last 30 days | 2.34 | 6.06 | | |
| Times participants went to the gym within the last 30 days | 2.55 | 6.74 | | |
|  |  |  | | |

*Note*. SD indicates standard deviation. Number of people refers to individuals outside one’s household.

**Table S2.**

| **Sure Amount** | **Risky Option** | **Expected Value for Risky Option** | **Trial Type** |
| --- | --- | --- | --- |
| $200 | 75% chance of $400 | 300 | Risky Advantageous |
| $100 | 50% chance of $250 | 125 | Risky Advantageous |
| $150 | 20% chance of 1000 | 200 | Risky Advantageous |
| $50 | 30% chance of $300 | 90 | Risky Advantageous |
| $50 | 80% chance of $150 | 120 | Risky Advantageous |
| $100 | 10% chance of $2000 | 200 | Risky Advantageous |
| $500 | 25% chance of $3000 | 750 | Risky Advantageous |
| $250 | 25% chance of $1300 | 325 | Risky Advantageous |
| $20 | 90% chance of $40 | 36 | Risky Advantageous |
| $100 | 90% chance of $150 | 135 | Risky Advantageous |
| $200 | 75% chance of $350 | 263 | Risky Advantageous |
| $400 | 50% chance of $1000 | 500 | Risky Advantageous |
| $200 | 75% chance of $265 | 199 | Risky Equal |
| $100 | 50% chance of $200 | 100 | Risky Equal |
| $150 | 20% chance of $750 | 150 | Risky Equal |
| $50 | 75% chance of $65 | 49 | Risky Equal |
| $50 | 70% chance of $75 | 49 | Risky Equal |
| $100 | 10% chance of $1000 | 100 | Risky Equal |
| $500 | 25% chance of $2000 | 500 | Risky Equal |
| $250 | 25% chance of $1000 | 250 | Risky Equal |
| $20 | 80% chance of $25 | 20 | Risky Equal |
| $100 | 90% chance of $110 | 99 | Risky Equal |
| $200 | 75% chance of $265 | 199 | Risky Equal |
| $400 | 50% chance of $800 | 400 | Risky Equal |
| $200 | 30% chance of $500 | 150 | Risky Disadvantageous |
| $100 | 50% chance of $150 | 75 | Risky Disadvantageous |
| $150 | 25% chance of $500 | 125 | Risky Disadvantageous |
| $50 | 60% chance of $75 | 45 | Risky Disadvantageous |
| $50 | 60% chance of $60 | 36 | Risky Disadvantageous |
| $100 | 20% chance of $450 | 90 | Risky Disadvantageous |
| $500 | 40% chance of $1100 | 440 | Risky Disadvantageous |
| $250 | 25% chance of $700 | 175 | Risky Disadvantageous |
| $22 | 70% chance of $25 | 17.5 | Risky Disadvantageous |
| $100 | 80% chance of $110 | 88 | Risky Disadvantageous |
| $200 | 75% chance of $225 | 168.75 | Risky Disadvantageous |
| $400 | 60% chance of $500 | 300 | Risky Disadvantageous |

*Note*. The order of whether the sure option or the risky option was presented as Option A or Option B was pseudo-randomized. Risky Advantageous means that the expected value for the risky option was higher than the sure amount. Risky Equal means that the expected value for the risky and sure options were nearly identical, and Risky Disadvantageous means that the expected value for the risky option was lower than the sure amount.

**Table S3.**

*Results of the Regression Model Predicting Appropriate Mask-Wearing Behavior*

| *Variable* | *Unstandardized Estimate* | | *S.E.* | *Standardized Estimate* | *P-Value* |
| --- | --- | --- | --- | --- | --- |
| Temporal Discounting | 25.562 | 5.701 | | 0.215 | <0.001 |
| Perceived Risk Difference | 13.975 | 2.533 | | 0.280 | <0.001 |
| Proportion of Risky Choices | -35.369 | 8.377 | | -0.199 | <0.001 |
| Data Collection Wave | 2.760 | 2.959 | | 0.040 | 0.355 |
| Political Affiliation | -0.878 | 1.693 | | -0.020 | 0.623 |
| COVID-19 Experience | -6.867 | 2.904 | | -0.100 | 0.018 |
| Negative Financial Consequences | -16.839 | 3.668 | | -0.231 | <0.001 |
| Age | -0.143 | 0.108 | | -0.056 | 0.191 |
| Education | -1.100 | 0.678 | | -0.071 | 0.112 |
| Income Level | 1.257 | 1.460 | | 0.036 | 0.418 |

*Note. R^2^= .356 (N=404, p<.001). The Proportion of Risky Choices refers to the average proportion of risky choices made in the Risky Choice Task. S.E. refers to Huber-White heteroscedasticity-robust standard errors for the unstandardized estimates.*

**Table S4.**

*Results of the Regression Model Predicting Number of Interpersonal Social Interactions*

| *Variable* | *Unstandardized Estimate* | *S.E.* |  | *Standardized Estimate* | *P-Value* |
| --- | --- | --- | --- | --- | --- |
| Temporal Discounting | -4.949 | 0.992 |  | -0.221 | <0.001 |
| Perceived Risk Difference | -1.815 | 0.518 |  | -0.193 | <0.001 |
| Proportion of Risky Choices | 6.214 | 1.830 |  | 0.185 | <0.001 |
| Data Collection Wave | -0.611 | 0.580 |  | -0.047 | 0.302 |
| Political Affiliation | 0.159 | 0.296 |  | 0.020 | 0.654 |
| COVID-19 Experience | 2.207 | 0.570 |  | 0.170 | <0.001 |
| Negative Financial Consequences | 1.849 | 0.622 |  | 0.135 | 0.003 |
| Age | 0.046 | 0.022 |  | 0.096 | 0.035 |
| Education | 0.469 | 0.134 |  | 0.160 | 0.001 |
| Income Level | -0.247 | 0.290 |  | -0.037 | 0.422 |

*Note. R^2^= .284 (N=404, p<.001). The Proportion of Risky Choices refers to the average proportion of risky choices made in the Risky Choice Task. S.E. refers to Huber-White heteroscedasticity-robust standard errors for the unstandardized estimates.*

**Table S5.**

*Results of the Regression Model Predicting Frequency of Non-Essential Social Activities*

| *Variable* | *Unstandardized Estimate* | *S.E.* | *Standardized Estimate* | *P-Value* |
| --- | --- | --- | --- | --- |
| Temporal Discounting | -35.192 | 4.914 | -0.282 | <0.001 |
| Perceived Risk Difference | -11.450 | 2.318 | -0.218 | <0.001 |
| Proportion of Risky Choices | 35.760 | 9.218 | 0.191 | <0.001 |
| Data Collection Wave | -7.668 | 2.688 | -0.106 | 0.009 |
| Political Affiliation | -1.371 | 1.456 | -0.030 | 0.434 |
| COVID-19 Experience | 8.662 | 2.726 | 0.120 | 0.002 |
| Negative Financial Consequences | 17.923 | 3.366 | 0.235 | <0.001 |
| Age | 0.097 | 0.105 | 0.037 | 0.364 |
| Education | 3.085 | 0.664 | 0.189 | <0.001 |
| Income Level | 1.036 | 1.477 | 0.028 | 0.496 |

*Note. R^2^= .438 (N=404, p<.001). The Proportion of Risky Choices refers to the average proportion of risky choices made in the Risky Choice Task. S.E. refers to Huber-White heteroscedasticity-robust standard errors for the unstandardized estimates.*

**Table S6.**

*Results of the Regression Model Predicting the Optimism Bias*

| *Variable* | *Standardized Estimate* | *S.E.* | *Standardized Estimate* | *P-Value* |
| --- | --- | --- | --- | --- |
| Temporal Discounting | -1.151 | 2.710 | -0.021 | 0.679 |
| Perceived Risk Difference | 1.882 | 1.103 | 0.082 | 0.105 |
| Proportion of Risky Choices | -12.890 | 3.946 | -0.158 | 0.002 |
| Data Collection Wave | 0.678 | 1.598 | 0.022 | 0.674 |
| Political Affiliation | -0.439 | 0.904 | -0.022 | 0.649 |
| COVID-19 Experience | 2.746 | 1.523 | 0.087 | 0.080 |
| Negative Financial Consequences | -3.256 | 1.684 | -0.098 | 0.057 |
| Age | 0.034 | 0.061 | 0.030 | 0.560 |
| Education | -1.339 | 0.417 | -0.188 | <0.001 |
| Income Level | 1.160 | 0.860 | 0.072 | 0.167 |

*Note. R^2^= .099 (N=404, p<.001). The Proportion of Risky Choices refers to the average proportion of risky choices made in the Risky Choice Task. S.E. refers to Huber-White heteroscedasticity-robust standard errors for the unstandardized estimates.*

**Table S7.**

*Results of the Regression Model Predicting Perceived Risk of Engaging in Activities in Public Settings Assuming that People Are Not Social Distancing*

| *Variable* | *Unstandardized Estimate* | | *S.E.* | *Standardized Estimate* | *P-Value* |
| --- | --- | --- | --- | --- | --- |
| Temporal Discounting | 0.348 | 0.178 | | 0.104 | 0.035 |
| Proportion of Risky Choices | -1.003 | 0.245 | | -0.200 | <0.001 |
| Data Collection Wave | -0.008 | 0.098 | | 0.004 | 0.936 |
| Political Affiliation | -0.326 | 0.058 | | -0.270 | <0.001 |
| COVID-19 Experience | 0.068 | 0.098 | | 0.035 | 0.474 |
| Negative Financial Consequences | -0.006 | 0.101 | | -0.003 | 0.955 |
| Age | -0.001 | 0.003 | | -0.020 | 0.689 |
| Education | 0.012 | 0.021 | | 0.027 | 0.601 |
| Income Level | 0.032 | 0.049 | | 0.032 | 0.533 |

*Note. R^2^= .126 (N=404, p<.001). The Proportion of Risky Choices refers to the average proportion of risky choices made in the Risky Choice Task. S.E. refers to Huber-White heteroscedasticity-robust standard errors for the unstandardized estimates.*

**Table S8.**

| *Correlational Analyses* |  |  |  |  |  |
| --- | --- | --- | --- | --- | --- |
|  | Appropriate Mask Wearing | Interpersonal  Social Interactions | Social Activities | Perceived Risk | Optimism Bias |
| Self-Reported Risk-Taking | -.37** | .35** | .47** | -.14** | -.17* |
| Worry | .18** | -.05 | .11* | .57** | -.11* |
| Stress from COVID-19 Uncertainty | .13* | -.03 | .04 | .40** | -.09 |
| *Note*. Interpersonal Social Interactions refers to the number of people participants had physical interactions with, and Social Activities indicates the number of times participants engaged in social gatherings or went to non-essential public places such as the mall, a dine-in restaurant, or a salon. Perceived Risk indicates the perceived risk of engaging in activities in public settings, such as plane travel, returning to in-person work and school, going to a restaurant, etc. | | | | | |

**indicates significance at the *p*<.001 level

*indicates significance at the *p*<.05 level

**Table S9.**

*Correlations between Prosocial Behavioral Intentions Questionnaire, Dictator Game, and Study Outcome Measures*

|  | Appropriate Mask Wearing | Interpersonal Social Interactions | Social Activities | Perceived Risk | Optimism Bias |
| --- | --- | --- | --- | --- | --- |
| PBIS | 0.053 | 0.046 | -0.005 | -0.036 | -0.133 |
| Dictator Game | -0.087 | 0.16* | 0.196* | 0.042 | -0.094 |

*Note.* PBIS refers to the Prosocial Behavioral Intentions Scale. The Dictator Game is defined as the amount participants opted to give to the recipient minus the amount kept for oneself. Higher scores reflect greater pro-sociality.

* indicates *p*<.05

**Table S10.**

*Multivariate Regression Analysis with all Independent and Dependent Variables.*

| Appropriate Mask-Wearing | *Estimate* | *S.E.* | *P-Value* | *R^2^* = 0.171 |
| --- | --- | --- | --- | --- |
| Temporal Discounting | 0.336 | 0.045 | <0.001 |  |
| Proportion of Risky Choices | -0.213 | 0.042 | <0.001 |  |
|  |  |  |  |  |
| Interpersonal Social Interactions |  |  |  | *R^2^* = 0.135 |
| Temporal Discounting | -0.296 | 0.043 | <0.001 |  |
| Proportion of Risky Choices | 0.195 | 0.044 | <0.001 |  |
|  |  |  |  |  |
| Non-Essential Social Activities |  |  |  | *R^2^* = 0.222 |
| Temporal Discounting | -0.388 | 0.031 | <0.001 |  |
| Proportion of Risky Choices | 0.236 | 0.045 | <0.001 |  |
|  |  |  |  |  |
| Optimism Bias |  |  |  | *R^2^* = 0.024 |
| Temporal Discounting | 0.011 | 0.048 | 0.815 |  |
| Proportion of Risky Choices | -0.154 | 0.038 | <0.001 |  |
|  |  |  |  |  |
| Perceived Risk |  |  |  | *R^2^* = 0.037 |
| Temporal Discounting | 0.128 | 0.050 | 0.010 |  |
| Proportion of Risky Choices | -0.132 | 0.038 | 0.001 |  |
|  |  |  |  |  |

*Note. Estimate refers to the unstandardized beta coefficients. S.E. refers to Huber-White heteroscedasticity-robust standard errors for the unstandardized estimates.*

**Risky Choice Task Procedure**

Instructions:

In this last part of the survey, you will choose between two different hypothetical options.  One of the options always offers a guaranteed amount of money.  If you choose that option, there's a 100% chance you that would win that amount.
The other option gives you a certain likelihood or chance of getting a larger amount but there's also a chance that you'll end up with nothing.

For example, you might be asked to choose between Options A and B below:
Option A:  Guaranteed $100
OR
Option B: 50% chance of $200 but also a 50% chance of $0

If you chose option A, that would mean you'd prefer to get $100 guaranteed for sure compared to option B.  If you chose Option B, that would mean you'd prefer a chance of getting a larger amount ($200) even though there's a 50% risk that you could end up with no money at all. 

Even though the choices below are hypothetical, please make choices as you would if you were making these decisions in real life. For example, you could imagine that a retail store offered you either a free $100 to use in their store or a lottery option where you have a 1 in 2 (50% chance) of getting a free $200 to use in their store but also a 50% chance that you get nothing at all.  What would you choose?

**Dictator Game Procedure to Assess Prosocial Behavior**

Instructions:

Imagine that a store is having a grand opening and is giving different amounts of money between $5 and $200 cash to the first 100 shoppers. You are shopper #99, and you get $100 cash.

Unfortunately, the store employee miscounted, and they do not have any money to give to shopper #100. The employee asks you if you would like to give some of your $100 to shopper #100. You do not know shopper #100, and it is very unlikely you will ever see them again in the future. How much money (if any) would you leave for shopper #100?


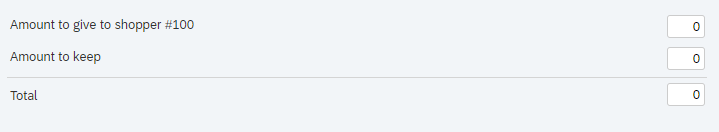

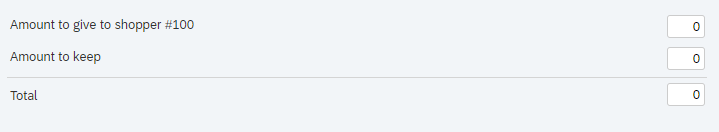


The outcome measure was defined as the amount participants opted to give to shopper #100 minus the amount they opted to keep for themselves. Higher scores reflect greater prosociality.

**Deviations from Open Science Framework (OSF) Pre-Registration**

Some deviations were made between the pre-registration and final study design. For full transparency, we document those changes below.

**Additions to Final Study Design**

First, to complement the mask-wearing dependent variable and broaden the scope of COVID-19 preventative behaviors to investigate, the two social distancing dependent variables (Interpersonal Social interactions and Non-Essential Social Activities) were added to the final study design. Before data collection began, we hypothesized that results for social distancing would mirror the results for mask-wearing. Specifically, we predicted that decreased social distancing would be associated with increased risk-taking behavior, decreased risk perception of COVID-19, and a tendency to prefer smaller immediate rewards over larger delayed rewards.

Secondly, the Perceived Difference variable (the difference in perceived risk when people are and are not social distancing) was added after all data was collected and was therefore a post-hoc modification. Using variables that were part of the pre-registration (Perceived Risk *while* social distancing and Perceived Risk *not* social distancing), this variable was created to demonstrate how effective participants perceived social distancing to be, and this variable was then used to predict COVID-19 preventative behavior (mask-wearing and social distancing).

**Changes to Final Study Design**

The path model was not pre-specified in the pre-registration and instead was added post-hoc as an exploratory analysis to show the relationships among both the independent and dependent variables. An additional change from the pre-registration to the final study was the stopping rule. The pre-registration stopping rule involved collecting data from approximately 200 participants over a two-week period in September 2020. Data was initially collected and analyzed in accordance with this stopping rule. The primary results showing the relationship between risky decision-making and temporal discounting with mask-wearing and social distancing for this initial data collection wave were largely the same as those reported in the current study. Upon review, given the uncertainty in beliefs and behavior surrounding COVID-19, we added a second wave of data collection in December 2020 in the final study design. Therefore, the second wave of data collection was a post-hoc addition to the final study. Moreover, the addition of the prosociality measures to the second wave was a further post-hoc addition.

The pre-registration also included the hypothesis that greater stress-related uncertainty due to COVID-19 would be associated with decreased risk-taking. While the results addressing this hypothesis are reported in the final study, the study focus was changed to decision-making and motivational factors that predict COVID-19 preventative behaviors, rather than both decision-making and emotional factors (stress and worry due to COVID-19) that affect compliance with COVID-19 prevention guidelines. We note that the study results do not support the pre-registration hypothesis. There was no association between stress-related uncertainty due to COVID-19 and risky choice, mask-wearing, or social distancing measures (ps>.50).

**Exclusions from Final Study Design**

The pre-registration stated that the study would examine ‘willingness to return to work’ which was defined as the percentage increase or decrease from one’s salary that participants would be willing to return to in-person work for during the pandemic. This data was collected, but it was not analyzed as part of the final study. The authors realized post-hoc that this variable was very different in scope from the decision-making focus of the paper and did not fit cohesively with the study purpose. Therefore, this variable was removed from the final study design.
