# Supplementary material for: Risk-taking unmasked: Using risky choice and temporal discounting to explain COVID-19 preventative behaviors
Source: PLoS One. 2021 May 13;16(5):e0251073. doi: 10.1371/journal.pone.0251073 (PMC8118306; doi:10.1371/journal.pone.0251073)
Supplement: Attachment — Submitted filename: Review - PONE-D-20-32678.pdf [file pone.0251073.s002.pdf]

Risk-Taking Unmasked: Using Risky Choice and Temporal Discounting to Explain COVID-19 Preventative Behaviors

The general idea of the paper is to analyze the correlation of risk preferences, temporal discounting, risk perception and measures of appropriate mask wearing, social distancing.

In order to do the analysis, the authors run an online experiment (n=225). Participants were recruited using MTurk (*N*=220) and the undergraduate subject pool at Clemson University (*N*=20).

The work is very well written and yields interesting results on the relations between different behaviors measures and the proper use of masks and social distancing. Despite this, it is a correlational study and some results should be considered with caution.

General comments

The paper analyzes the relationship between COVID-19 preventative behaviors and individual differences in four classic judgment and decision-making constructs. But, the correct use of masks and compliance with social distancing can be seen also as a collective action problem, where there are other hypotheses that can explained how and why people cooperates. Also, the COVID could have a direct impact on risk, delayed discounting and selfishness (see Brañas et al., 2020a; Adena and Harke, 2020). At the end, the results that authors can be seen is that people became more selfish, impatient or risk averse as a response to this situation, and they become even more as the day passed in the time window that did the survey. Probably, author need to add a paragraph with this discussion and might controlled for days fixed effect in the regression analysis.

Also, the independent variables used do not reflect directly compliance with COVID-19 prevention guidelines. Preventive measures are public knowledge, so many people can answer what is socially desirable and do not necessarily reveal their true intention. Probably authors need to discuss the social desirability bias in their hypotheses.

Specific comments

a) They talked about the study's limitations; they need to analyze how representative is the sample to the standard US population. With 225 observations, probably is not representative and the external validity of the results is very restricted.
b) Despite the power calculations made, the number of observations is low for a Mturk sample. However, the design is very good and the results are very interesting, so authors should think about redoing the experiment with a larger sample. It is not necessary to do it in Mturk. Jorrat (2020) suggests a procedure

to do online experiments in a short time and achieve a high number of observations.

c) Another interesting independent variable to analyze could be the difference between the perceived risk of the different activities with and without social distancing. This could be a measure of how effective people think social distancing is.

d) Authors need to discuss about why hypothetical time a risk experimental measures are a good proxy of incentivized ones. These papers study this experimental question:

   Brañas-Garza, P., Jorrat, D., Espín, A. M., & Sanchez, A. (2020). Paid and hypothetical time preferences are the same: Lab, field and online evidence. arXiv preprint arXiv:2010.09262.

   Brañas-Garza, P., Estepa Mohedano, L., Jorrat, D., Orozco, V., & Rascon-Ramirez, E. (2020). To pay or not to pay: Measuring risk preferences in lab and field.

   Falk, A., Becker, A., Dohmen, T. J., Huffman, D., and Sunde, U. (2015). The preference survey module: A validated instrument for measuring risk, time, and social preferences. IZA Discussion Paper.

e) A regression analyses with all the dependent variables is need it. Authors can made different specifications and add each of the four variables separately and other specifications with all the variables. Authors also need to put the regressions tables in the supplementary materials.

References:

Adena, M. & Harke, J, (2020). COVID-19 and pro-sociality: the effect of pandemic severity and increased pandemic awareness on charitable giving. Mimeo.

Branas-Garza, P., Jorrat, D. A., Alfonso, A., Espin, A. M., García, T., & Kovarik, J. (2020). Exposure to the Covid-19 pandemic and generosity. https://doi.org/10.31234/osf.io/6ktuz

Jorrat, D. A. (2020). Recruiting experimental subjects using WhatsApp. https://doi.org/10.31234/osf.io/6vgec
