# Supplementary material for: Risk-taking unmasked: Using risky choice and temporal discounting to explain COVID-19 preventative behaviors
Source: PLoS One. 2021 May 13;16(5):e0251073. doi: 10.1371/journal.pone.0251073 (PMC8118306; doi:10.1371/journal.pone.0251073)
Supplement: Attachment — Submitted filename: Report 2.pdf [file pone.0251073.s004.pdf]

The present version of the paper is improved and addresses most of the issues that the other reviewer and I had raised. I also accept that there may be differences in methods and approaches between psychology and economics, which should be toned down in an interdisciplinary journal like PLOS-1. Nonetheless, the authors do choose economic theory to justify their methods, and should then make sure that their approach is correct. Most importantly, I am still unclear on the nature of the econometric model the authors use. I would then recommend the authors to address the following issues:

1) The authors inserted the following statement to explain their approach concerning the measurement of risk aversion (pp. 4-5):

Choosing risky options with higher expected utility is economically advantageous, while choosing risky options that have a lower expected utility than a safe option is maladaptive and can often lead to sub-optimal decision-making (Weller, Levin, & Bechara, 2010; von Neumann & Morgenstern, 1944).

I find this statement confusing and factually wrong. I suspect that the authors confuse expected utility with the expected value of a gamble (i.e. a risky choice). Otherwise, taking this sentence literally would entail that individuals would choose "risky options that have a lower expected utility than a safe option". Since utility is unobservable, the principle of revealed preferences by von Neumann and Morgestern (cited) – after the rationalization by Savage (1954) -, states that, precisely for this reason, we can deduct the expected utility function from actual choices that people make. Observing individuals choosing lotteries having lower expected utility than a safe option is then by construction impossible within the theory. This is why I guess that the authors meant "expected value" rather than expected utility, as also suggested by the language they use in other parts of the paper (e.g. figures' captions). Even so, their statement is factually incorrect. While saying that lotteries with higher expected value are economically advantageous is a platitude, saying that "*choosing risky options having a lower expected *value* than a safe option is maladaptive and can often lead to sub-optimal decision-making*" goes against cross-country experimental evidence finding that economically richer countries are characterized, on average, by lower, rather than higher, risk tolerance (Falk et al., 2018; Bouchouicha, R., & Vieider, 2019). Admittedly, this finding only holds after controlling for other psychological traits (see Table 9 in Falk et al., 2018), while an opposite relation between risk tolerance and income holds *within* country (l'Haridon & Vieider, 2019). Nevertheless, this evidence is enough to make the authors' statement basically unfounded. Moreover, it is perfectly acceptable, I would dare say from a psychological point of view, that individuals prefer a safe option to a gamble, be it advantageous or disadvantageous, because it is perfectly acceptable that for most individuals well-being (or utility) is decreased by risk. This should not be seen as either maladaptive or sub-optimal, as it is in fact a rather widespread characteristic of individual preferences.

In sum, I find the authors' justification of their multi-level approach used in their econometric analysis to be flawed theoretically and empirically. I would suggest the authors to drop any reference to expected utility theory. I would instead ask the authors to provide a different theoretical justification of the reason why we should consider choices in the disadvantageous, equal, or advantageous domain, as distinct from one another, possibly relying on other studies doing so.

2) Even if the reader can now have a clear view of how the measurement of risk-taking behaviour was carried out, thanks to Table S2 in the Appendix, I still find their econometric model unclear.

Have the authors taken the three following measures for each individual: [proportion of risky choices taken in the disadvantageous domain; proportion of risky choices in the “equal” domain; proportion of risky choices in the advantageous domain]? This is what appears from the descriptive statistics. If this is the case, this should be stated clearly. But then, what is the purpose of including what appear to be interaction terms between the “level” of the variables, as defined above, and the proportion of risky choices? This is what appears in Table S3 in the Appendix, while the caption seems to indicate the lack of inclusion of interaction terms:

Equal EV Gamble X Equal Risky Choices indicates proportion of equal EV risky choices and Disadv. EV Gamble X Disadv. Risky Choices refers to proportion of disadvantageous EV risky choices.

But then, why do the authors include these five variables in the model?

Risky Choice EV Type
Equal EV Gamble
Disadvantageous EV Gamble
Equal EV Gamble X Equal Risky Choices
Disadv. EV Gamble X Disadv. Risky Choices

By reading that the authors used a repeated measure model, my understanding was that the authors organized their data in panel format, with the individual as the cross-sectional unit and the three decisions at the three levels as the “longitudinal” variable. But having these interaction terms in the model (assuming they are interaction terms) makes me think that the authors used instead a pooled model? And in any case, what is the point of having an interaction term between the different levels? Are we at all interested in knowing that participants made riskier decisions in the advantageous domain? This seems obvious, and I have not seen this result being reported. In conclusion, I think it is necessary that the authors provide more details of their econometric approach, written down with equations to avoid any misunderstanding. I would also appreciate if the authors could make available their data and codes they used in their analysis. This is requested in any case by publication in PLOS1.

The authors should also report in Table S3 the total number of observations, and the observations by cluster (three, if my interpretation is correct). Since observations are clustered at the individual level, the best practice is to use heteroschedasticity-robust standard errors clustered at this level.

3) As could have been expected, only a minority of people chose risky options in the disadvantageous and equal domain, while more chose the risky option in the advantageous domain. The authors discuss this aspect in the discussion section:

“Given that the risk-taking findings were specific to equal and disadvantageous expected value contexts, it should be noted that some participants chose the safe option on all trials. Therefore, the relationship between risk-taking and compliance behavior may be driven by a subsample of the sample, which may limit the generalizability of the findings” .

This statement sounds unnecessarily reticent. Rather than saying that the results are driven by "some participants", the authors should quantify how many participants chose the safe option in all cases. I would expect a more extensive discussion of the fact that the result is driven by only a minority of participants. This is not at all uncommon in statistical analysis. Moreover, for social phenomena like mask-wearing, the behavior of a minority individuals, maybe as low as 10% of the population, may be enough to tip the social equilibrium from one of general compliance to one of general non-compliance. Therefore, I do not think that even if the result was driven by a minority of participants would detract from the general interest of the paper.

4) Even if the above clarifications are necessary in my opinion, I still struggle to see the need for breaking down the observations by domain (advantageous, equal, disadvantageous gambles). What is this regression is telling us? Is it really telling us that people who are more risk tolerant, as measured in the "cups task", wear the mask less frequently? This would seem the type of analysis to address the research question of the paper. Instead, my impression is that this regression is telling us something (partially) different, that is, that among all of the risky gamble choices that participants made, those that are more predictive of mask-wearing behavior are those made in the disadvantageous and equal domains. For example, suppose that Participant P1 chose all risky options in the advantageous domain, and all safe options in the equal and disadvantageous domain. Suppose that Participant P2 chose all safe options in the advantageous domain and in the equal domain, while choosing a quarter of risky choices in the disadvantageous domain. The econometric model would predict that P1 should wear a mask in public, while P2 should not. But can we infer in any meaningful sense that this kind of choice captures that P2 is more risk tolerant than P1? Or is this finding relevant purely on methodological grounds, rather than to associate individual psychological traits to individual real-life behavior?
   To me it would seem more natural to consider one measure of risk tolerance for each individual defined over all the possible set of lotteries. Even if more sophisticated measures would be possible, an obvious candidate would be to use the proportion of risky choices over the whole set of 36 choices. This measure should be used as the main predictor in a regression analogous to the one reported in Table S3. Looking at the correlations in Table 4, it would appear that this variable would turn out as a significant predictor of mask-wearing. The authors could subsequently break down the analyses by domain, as they presently do.
5) I note that the authors went at great length in addressing the comment that pro-sociality was a possible confound in the theoretical model. I agree with the authors that giving too much attention to this aspect would detract from the main focus of the paper. Nonetheless, since both the other reviewer and I had the same concern, it is quite likely that other readers would as well. I would then recommend the authors to report their analysis concerning pro-sociality in the Appendix and mention the result in the main paper. I would also stress that it is obvious that the list of possible explanatory variables is potentially infinite, and nobody in their right mind could ask to incorporate all of them in the analysis. Nonetheless, pro-sociality is such an obvious possible motivator in the current context, that many would feel that not including could jeopardize the interpretation of the results.

References

Bouchouicha, R., & Vieider, F. M. (2019). Growth, entrepreneurship, and risk-tolerance: a risk-income paradox. *Journal of Economic Growth*, *24*(3), 257-282.

Falk, A., Becker, A., Dohmen, T., Enke, B., Huffman, D., & Sunde, U. (2018). Global evidence on economic preferences. *The Quarterly Journal of Economics*, *133*(4), 1645-1692.

l'Haridon, O., & Vieider, F. M. (2019). All over the map: A worldwide comparison of risk preferences. Quantitative Economics, 10(1), 185-215.

Savage, L. J., 1954, The Foundations of Statistics, Wiley, New York. Second revised edition, Dover Publications, New York, 1972.
