# Supplementary material for: Risk-taking unmasked: Using risky choice and temporal discounting to explain COVID-19 preventative behaviors
Source: PLoS One. 2021 May 13;16(5):e0251073. doi: 10.1371/journal.pone.0251073 (PMC8118306; doi:10.1371/journal.pone.0251073)
Supplement: Attachment — Submitted filename: ReviewerComments2.docx [file pone.0251073.s005.docx]

The present version of the paper is improved and addresses most of the issues that the other reviewer and I had raised. I also accept that there may be differences in methods and approaches between psychology and economics, which should be toned down in an interdisciplinary journal like PLOS-1. Nonetheless, the authors do choose economic theory to justify their methods, and should then make sure that their approach is correct. Most importantly, I am still unclear on the nature of the econometric model the authors use. I would then recommend the authors to address the following issues:

1) The authors inserted the following statement to explain their approach concerning the

measurement of risk aversion (pp. 4-5):

*Choosing risky options with higher expected utility is economically advantageous, while choosing risky*

*options that have a lower expected utility than a safe option is maladaptive and can often lead to*

*sub-optimal decision-making (Weller, Levin, & Bechara, 2010; von Neumann & Morgenstern, 1944).*

I find this statement confusing and factually wrong. I suspect that the authors confuse expected utility with the expected value of a gamble (i.e. a risky choice). Otherwise, taking this sentence

literally would entail that individuals would choose “risky options that have a lower expected utility

than a safe option”. Since utility is unobservable, the principle of revealed preferences by von

Neumann and Morgestern (cited) – after the rationalization by Savage (1954) -, states that, precisely

for this reason, we can deduct the expected utility function from actual choices that people make.

Observing individuals choosing lotteries having lower expected utility than a safe option is then by

construction impossible within the theory. This is why I guess that the authors meant “expected

value” rather than expected utility, as also suggested by the language they use in other parts of the

paper (e.g. figures’ captions). Even so, their statement is factually incorrect. While saying that

lotteries with higher expected value are economically advantageous is a platitude, saying that

“choosing risky options having a lower expected *value* than a safe option is maladaptive and can

often lead to sub-optimal decision-making” goes against cross-country experimental evidence finding

that economically richer countries are characterized, on average, by lower, rather than higher, risk

tolerance (Falk et al., 2018; Bouchouicha, R., & Vieider, 2019). Admittedly, this finding only holds

after controlling for other psychological traits (see Table 9 in Falk et al., 2018), while an opposite

relation between risk tolerance and income holds within country (l'Haridon & Vieider, 2019).

Nevertheless, this evidence is enough to make the authors’ statement basically unfounded. Moreover, it is perfectly acceptable, I would dare say from a psychological point of view, that

individuals prefer a safe option to a gamble, be it advantageous or disadvantageous, because it is

perfectly acceptable that for most individuals well-being (or utility) is decreased by risk. This should

not be seen as either maladaptive or sub-optimal, as it is in fact a rather widespread characteristic of

individual preferences.

In sum, I find the authors’ justification of their multi-level approach used in their econometric

analysis to be flawed theoretically and empirically. I would suggest the authors to drop any reference

to expected utility theory. I would instead ask the authors to provide a different theoretical

justification of the reason why we should consider choices in the disadvantageous, equal, or

advantageous domain, as distinct from one another, possibly relying on other studies doing so.

*To correct this issue in the Introduction, we have reframed this paragraph by removing all references to expected utility. We conceptual adaptive or optimal decision-making in terms of reward maximation, which depends on sensitivity to expected values (not utility, in accordance with what the comment above describes), rather than utility or individual preference. This conceptualization is in line with the 7,000+ studies that have relied on tasks like the Cups Task and Iowa Gambling Task (Bechara et al., 1994) and the Expectancy Valence model of behavior on these tasks (Yechiam et al., 2005; Worthy, Pang, & Byrne, 2013), which consistently shows that greater sensitivity to expected values is associated with greater reward maximization. Indeed, the Cups Task was developed in collaboration with Bechara, who developed the IGT.*

*We are a bit unclear on the comparisons between the references cited on the relationship between income and risk tolerance and the assumption that choosing higher expected values in classic risky choice decision-making paradigms leads to adaptive decision-making (i.e., decision-making that maximizes reward). We have statistically controlled for income by including income level as a covariate in the regressions. Moreover, behavior that leads to reward maximization is quite different from the demographic variable of income, which is largely a product of one’s life circumstances, privilege (i.e., white privilege), and inequality in access to resources.*

*We have updated the paragraph to instead rely on the same framework as the one used in the seminal Cups Task paper: sensitivity to expected values. We replace the sentence described above with the following (p. 5): “Choosing options with higher expected values reflects increased sensitivity to differences in expected value between choice options [7, 8]. As evidenced by performance on decision-making paradigms such as the Iowa Gambling Task [9, 10] and the Cups Task [7,8], this increased sensitivity can lead to reward maximization”. In other words, choosing the risky choice in advantageous expected value contexts but not disadvantageous expected value contexts reflects increased reward sensitivity and is likely to lead to reward maximation. Using this task allows us to assess both risk-taking behavior (overall performance on the Cups Task) and sensitivity to expected values (varying behavior in different expected value contexts). This explanation was already expressed in the Discussion (p. 30, top), but we failed to make this information clear in the Introduction originally. However, given the comments below, we’ve toned down the emphasis on risky choice in these varying contexts.*

2) Even if the reader can now have a clear view of how the measurement of risk-taking behaviour

was carried out, thanks to Table S2 in the Appendix, I still find their econometric model unclear.

Have the authors taken the three following measures for each individual: [proportion of risky

choices taken in the disadvantageous domain; proportion of risky choices in the “equal” domain;

proportion of risky choices in the advantageous domain]? This is what appears from the descriptive statistics. If this is the case, this should be stated clearly. But then, what is the purpose of including what appear to be interaction terms between the “level” of the variables, as defined above, and the proportion of risky choices? This is what appears in Table S3 in the Appendix, while the caption seems to indicate the lack of inclusion of interaction terms:

Equal EV Gamble X Equal Risky Choices indicates proportion of equal EV risky choices and Disadv. EV

Gamble X Disadv. Risky Choices refers to proportion of disadvantageous EV risky choices.

But then, why do the authors include these five variables in the model?

Risky Choice EV Type

Equal EV Gamble

Disadvantageous EV Gamble

Equal EV Gamble X Equal Risky Choices

Disadv. EV Gamble X Disadv. Risky Choices

By reading that the authors used a repeated measure model, my understanding was that the authors

organized their data in panel format, with the individual as the cross-sectional unit and the three

decisions at the three levels as the “longitudinal” variable. But having these interaction terms in the

model (assuming they are interaction terms) makes me think that the authors used instead a pooled

model? And in any case, what is the point of having an interaction term between the different levels?

Are we at all interested in knowing that participants made riskier decisions in the advantageous

domain? This seems obvious, and I have not seen this result being reported. In conclusion, I think it is

necessary that the authors provide more details of their econometric approach, written down with

equations to avoid any misunderstanding. I would also appreciate if the authors could make available their data and codes they used in their analysis. This is requested in any case by publication in PLOS1. The authors should also report in Table S3 the total number of observations, and the observations by cluster (three, if my interpretation is correct). Since observations are clustered at the individual level, the best practice is to use heteroschedasticity-robust standard errors clustered at this level.

*We acknowledge that the presentation of the regressions was unclear, and we have now added a “Data Analysis” subsection to the paper to clarify the statistical approach for the regressions*. *Originally, the proportion of risky choices was included as a main effect, Expected Value Level (dummy coded) was also a main effect, and then the Proportion of Risky Choices X Expected Value Level interaction term was included, which allowed for assessing whether risky choices in the advantageous, disadvantageous, or equal gamble domains were predictive of each dependent variable. There were 5 terms included in the tables because MPlus and R (the statistical analysis tools we use), have options to combine the main effects and simple contrasts to identify the locus of the interaction into one table, which can be more informative. The rationale for including the different levels was to show the contexts in which participants made risky choices—essentially* *does all risky choice or just risky choices that leads to lower expected values (i.e., disadvantageous) affect mask-wearing and social distancing? To answer this question, it is important methodologically to include advantageous risky choices as a comparison group.*

*However, based on these comments, we can see where this approach may ‘muddy the waters’ and make the analyses unnecessarily complex without adding substantial knowledge depth. As such, we have now removed the analysis by EV level from the regression models. Now, average proportion of risky choices (averaged across advantageous, disadvantageous, and equal contexts) alone is included in the model, and multiple regressions are used. We have also added the regression equation. This information is now explained in the new Data Analysis sub-section (p. 18).*

*Furthermore, in the description of the risky choice task, we now state “the average proportion of risky gambles across all gambling types was computed for regression analyses and used as the primary analysis variable for this task. Additionally, following previous research using the Cups Task and similar risky choice paradigms [8, 63—70], the proportion of risky choices for each gamble type (risky advantageous, risky equal, and risky disadvantageous) was computed and used in follow-up analyses; this provides further information about whether sensitivity to expected values, as reflected by differential risk-taking in advantageous compared to disadvantageous decision contexts, influences the outcome variables” (p. 16, bottom & p. 17, top).*

*The data is available; we included the link in the PLoS One submission questions but didn’t realize this information was not sent to reviewers; we apologize for our misunderstanding. The link to the data is here:*

*<https://osf.io/xy6aj/?view_only=1360495284da453baac8df96c7a732d1>*

*Upon manuscript acceptance, we will remove the ‘view-only’ link and the link will be* [*https://osf.io/xy6aj/*](https://osf.io/xy6aj/)*. This link has now been added to the manuscript. We are hesitant to make the data available outside of a view-only link before manuscript acceptance because we have had our data stolen by an outside research group in the past.*

*We have now added the number of observations/participants and heteroscedasticity-robust standard errors (Huber-White standard errors) to Tables S3 – S7 in addition to the R^2^-value and p-value for the omnibus test.*

*In terms of addressing the specific statistical concerns, we note that our analytical expertise is rooted in training from experimental psychology and computer science. Therefore, we are unfamiliar with some of the terminology referred to (e.g., econometric model, cross-sectional units, pooled models, etc.), as this lies outside of our field. We mention this simply to acknowledge that we may use different terms to refer to similar concepts/approaches that exist in other fields, such as economics.*

3) As could have been expected, only a minority of people chose risky options in the

disadvantageous and equal domain, while more chose the risky option in the advantageous

domain. The authors discuss this aspect in the discussion section:

*“Given that the risk-taking findings were specific to equal and disadvantageous expected value contexts, it should be noted that some participants chose the safe option on all trials. Therefore, the relationship between risk-taking and compliance behavior may be driven by a subsample of the sample, which may limit the generalizability of the findings”.*

This statement sounds unnecessarily reticent. Rather than saying that the results are driven by

“some participants”, the authors should quantify how many participants chose the safe option in all cases. I would expect a more extensive discussion of the fact that the result is driven by only a

minority of participants. This is not at all uncommon in statistical analysis. Moreover, for social

phenomena like mask-wearing, the behavior of a minority individuals, maybe as low as 10% of the

population, may be enough to tip the social equilibrium from one of general compliance to one of

general non-compliance. Therefore, I do not think that even if the result was driven by a minority of

participants would detract from the general interest of the paper.

*We have now added the percentage of participants who chose the safe option in all cases to the Results section (p. 20, bottom). In particular, we state: “In terms of risky decision-making, 17.08% of participants chose the safe option on all trials. By gambling context, 19.55% of participants chose the safe option in all advantageous expected value contexts, 46.78% chose the safe option in all equal expected value contexts, 57.43% chose the safe option in all disadvantageous contexts.”*

*We have updated the Discussion to include a more extensive discussion of this finding (p.30, middle). Specifically, we have added the following text: “It should be noted that the average percentage of risky choices across all participants in equal and disadvantageous contexts was low—less than 20%. Moreover, over half (57%) of participants chose the safe option on all disadvantageous trials, and 47% chose the safe option on all equal expected value trials. The relationship between risk-taking and compliance behavior appears to be driven by a minority of participants, which may limit the generalizability of the findings. Nevertheless, the behavior of even a minority of individuals can have substantial impacts during a pandemic. Recent estimates indicate that approximately 10% of infected individuals account for 80% of COVID-19 transmission [75]. Though a minority of individuals engage in equal and disadvantageous risky decision-making, the corresponding decrease in compliance with mask-wearing and social distancing observed in this study could have wide-ranging consequences for spreading COVID-19.*

4) Even if the above clarifications are necessary in my opinion, I still struggle to see the need for breaking down the observations by domain (advantageous, equal, disadvantageous gambles).

What is this regression is telling us? Is it really telling us that people who are more risk tolerant,

as measured in the “cups task”, wear the mask less frequently? This would seem the type of

analysis to address the research question of the paper**.** For example, suppose that Participant P1 chose all risky options in the advantageous domain, and all safe options in the equal and disadvantageous domain. Suppose that Participant P2 chose all safe options in the advantageous domain and in the equal domain, while choosing a quarter of risky choices in the disadvantageous domain. The econometric model would predict that P1 should wear a mask in public, while P2 should not. But can we infer in any meaningful sense that this kind of choice captures that P2 is more risk tolerant than P1? Or is this finding relevant purely on methodological grounds, rather than to associate individual psychological traits to individual real-life behavior?

To me it would seem more natural to consider one measure of risk tolerance for each individual

defined over all the possible set of lotteries. Even if more sophisticated measures would be

possible, an obvious candidate would be to use the proportion of risky choices over the whole

set of 36 choices. This measure should be used as the main predictor in a regression analogous

to the one reported in Table S3. Looking at the correlations in Table 4, it would appear that this

variable would turn out as a significant predictor of mask-wearing. The authors could

subsequently break down the analyses by domain, as they presently do.

*We appreciate the feedback on this point. To briefly explain our original rationale, the original regression was intended to gauge whether individuals’ risky decisions on the Cups Task (in addition to other factors) relates to their decisions regarding mask-wearing and social distancing. The risk level (advantageous, disadvantageous, and equal expected value) for this* *task is typically included as a modifier—simply to provide more specific information about the contexts in which people make or do not make risky choices (disadvantageous vs. equal, etc.). Those that make riskier choices in advantageous contexts more than disadvantageous contexts are considered more sensitivity to changes in expected value [7, 8]. The task and analytical approach were intended to capture this context variability because* *risk-taking is not a static ‘trait’ but varies within a single individual based on domain, context, and time (Weber et al., 2002), while psychological traits are defined as consistent over time and context. We realize that this information was not conveyed clearly in the previous revision.*

*Based on the feedback, we have now revised the regressions for each outcome measure to include a single predictor of risky decision-making behavior, which is defined as the proportion of risky choices over the whole set of choices as suggested. After reporting the results of the primary regressions, we report the results of separate follow-up tests that break down the analyses by gambling domain. The results showed that both risky decision-making behavior and temporal discounting predicted decreased mask-wearing and social distancing behavior. There were changes in the significance level for some of the covariates, and this information has been updated throughout the results and discussion. We have also now included some brief additional information to better explain why the different contexts (advantageous, disadvantageous, and equal) were examined as part of the study (p. 5, top). The corresponding tables have also now been updated. Overall, this updated approach led to more straightforward results that we think may be clearer and more useful for a broad audience like the PLoS One readership to interpret.*

*Weber, E. U., Blais, A. R., & Betz, N. E. (2002). A domain‐specific risk‐attitude scale: Measuring risk perceptions and risk behaviors. Journal of behavioral decision making, 15(4), 263-290.*

5) I note that the authors went at great length in addressing the comment that pro-sociality was a

possible confound in the theoretical model. I agree with the authors that giving too much

attention to this aspect would detract from the main focus of the paper. Nonetheless, since both

the other reviewer and I had the same concern, it is quite likely that other readers would as

well. I would then recommend the authors to report their analysis concerning pro-sociality in

the Appendix and mention the result in the main paper. I would also stress that it is obvious that

the list of possible explanatory variables is potentially infinite, and nobody in their right mind

could ask to incorporate all of them in the analysis. Nonetheless, pro-sociality is such an obvious

possible motivator in the current context, that many would feel that not including could

jeopardize the interpretation of the results.

*We have now added the description of the prosociality measures to the Methods section (p. 17). We then elaborate on the methodological details and report the results in the Supplementary Material and mention the correlational results in the Results section of the main paper (p. 29) and in the Discussion (p. 34). The sentence stating that an exhaustive list of possible influences on COVID-19 compliance has also been removed.*

References

Bouchouicha, R., & Vieider, F. M. (2019). Growth, entrepreneurship, and risk-tolerance: a risk-income

paradox. Journal of Economic Growth, 24(3), 257-282.

Falk, A., Becker, A., Dohmen, T., Enke, B., Huffman, D., & Sunde, U. (2018). Global evidence on

economic preferences. The Quarterly Journal of Economics, 133(4), 1645-1692.

l'Haridon, O., & Vieider, F. M. (2019). All over the map: A worldwide comparison of risk

preferences. Quantitative Economics, 10(1), 185-215.

Savage, L. J., 1954, The Foundations of Statistics, Wiley, New York. Second revised edition, Dover

Publications, New York, 1972.
